# Unexpected High Need for Genetic Testing in Rheumatology: A Cross-Sectional Cohort Study

**DOI:** 10.3390/genes14101858

**Published:** 2023-09-24

**Authors:** Lukas Kampik, Michael Schirmer

**Affiliations:** Department of Internal Medicine, Clinic II, Medical University of Innsbruck, 6020 Innsbruck, Austria; lkskmpk@gmail.com

**Keywords:** rheumatology, genetic, diagnosis, pharmacogenetic, prognosis, management

## Abstract

Background: Genetic testing may provide information for diagnostic, prognostic and pharmacogenetic purposes. The PREPARE study recently showed that the number of clinically relevant adverse drug reactions could be reduced via genotype-guided treatment. The aim of this work was to assess the relevance of genetic testing and its actual use in consecutive rheumatic outpatients. Methods: A retrospective cross-sectional analysis was performed with data from a prospectively designed observational project with outpatients consecutively recruited from a university clinic of rheumatology. Results: In this cohort of 2490 patients, the potential need for genetic testing is immense, with 57.3% of patients having the potential to benefit from genetic testing according to their diagnosis and treatment and 53.3% of patients with actually performed genetic testing for diagnostic, prognostic or pharmacogenetic purposes. In detail, patients would potentially benefit from genetic testing especially for therapeutic (28.0%) and diagnostic (26.9%) purposes. Genetic testing was performed for diagnostic purposes in 51.6% of subjects, for pharmacogenetic purposes in 3.7% and for prognostic purposes in 0.1%. The ratio between the number of patients who had had tests performed to those with a potential need for genetic testing decreased with age, from 127.1% for 20 to <30-year-old patients to 46.1% for 80 to <90-year-old patients. Pharmacogenetic testing was only performed for disease-related medications. Conclusions: Genetic testing is frequently needed in patients with rheumatic diseases. The value of pharmacogenetic testing is certainly underestimated, especially in case of medications for comorbidities.

## 1. Introduction

Genetic testing is performed to identify variant mutations of DNA, which can be clinically relevant for diagnostic, pharmacogenetic and prognostic purposes. Thus, genetic testing may be relevant for pharmacological decisions as well as making diagnosis and prognostic consultations. This applies not only to personalized medicine in hematology and oncology but also becomes more and more relevant for patients with rheumatic diseases. Susceptibility to rheumatic diseases such as spondyloarthritis (SpA), rheumatoid arthritis (RA) or psoriatic arthritis (PsA) is attributable largely to the genomic background of patients, with heritability estimates ranging from 50% for osteoarthritis to >90% for PsA [1].

Over the last decade, molecular diagnostic testing in a symptomatic individual has become increasingly sophisticated. While genetic testing was initially carried out for only one or a few mutations, it is now possible to apply cheaper, faster and more accurate whole-exome sequencing and whole-genome sequencing techniques [2]. Therefore, today even the identification of DNA variants in unselected individuals can be considered predictive of latent disease risk. This identification, known as DNA-based screening, is a new approach to health screening [3]. The goal of such genetic screening is to identify individuals who are at sufficient risk for a particular disease or side effect of pharmaceutical treatment and could benefit from either further testing or direct preventive measures. Of note is that such DNA-based screening is not the same as DNA-based diagnostic testing, as DNA-based testing is performed as part of a diagnostic effort in patients with an increased likelihood of a disease related to a positive genetic test result, based on signs, symptoms, physical examination, other diagnostic tests or family history prior to the test [3].

DNA sequencing for DNA variants may provide important information on possible disease risks, not only for cardiovascular and neurological diseases but also for rheumatic diseases [4]. Specific genes summarized as ‘tier 1 genomic applications’ are regarded as the core list to be considered for clinical genetic screening [5]. However, a positive test from DNA screening does not immediately imply the diagnosis of the questionable ‘health problem’. In rheumatology, a patient with HLA-B27 positivity but without clinical evidence of SpA has a higher disease risk but still does not fulfill the diagnosis of SpA. Also, a negative screening result does not exclude the possible diagnosis of SpA. If DNA-based screening is intended to improve population health, it must be combined with effective risk-reducing clinical counseling and care [3]. Only pathogenic and likely pathogenic results should be reported to the patient to ensure risk-mitigating clinical care. Observed genetic variants without sufficient evidence for clinical diagnosis should be excluded from genetic counseling [3]. Diagnostic genetic testing is most relevant for the diagnosis of SpA (*HLA-B27*), Behçet’s disease (*HLA-B51*) and rare diseases such as fever syndromes, including familial Mediterranean fever (*FMF*) (*familial Mediterranean fever gene* (*MEVF*)) amongst others. HLA-B27*06 and HLA-B27*09, however, are not associated with SpA [6]. Still, genetic results alone are unspecific for rheumatic diagnosis and only relevant in combination with specific clinical, laboratory or imaging findings.

Prognostic testing could be helpful for education of the patients but is not routinely recommended or performed in rheumatic patients. Only in some diseases, such as RA, may genetic results such as the *HLA-DRB* locus (‘shared epitopes’) be considered prognostic factors. It is estimated that the genetic contribution to RA pathogenesis is about 60% [7]. HLA-DR*01 and *04 are associated with a more aggressive course of RA, whereas other *HLA-DRB1* alleles appear to be more protective. Interestingly, the association between *HLA-DRB1* ‘shared epitopes’ and RA risk applies to patients with antibodies directed against citrullinated proteins (ACPA), whereas the protective alleles such as DRB1*03 are associated with ACPA-negative RA [7,8]. Thus, prognostic genetic testing is no longer considered necessary for routine patient consultation on prognosis [9,10].

Concerning pharmacogenetic testing, the recently published study ‘Preemptive Pharmacogenomic Testing for Preventing Adverse Drug Reactions’ (PREPARE) showed that the number of clinically relevant adverse drug reactions could be reduced via genotype-guided treatment based on a 12-gene pharmacogenetic panel [11]. It is estimated that 20–30% of the variable drug effects are due to genetic factors [12]. These genes then may influence drug transformation, pharmacokinetics and pharmacodynamics, possibly leading to adverse events [12,13]. Also, pharmacogenetics may lead to low or absent treatment response, although the exact causes of drug resistance are sometimes unclear [13]. Nevertheless, most medications are currently prescribed without the consideration of pharmacogenetic information [14], as the strength of evidence required for the clinical implementation of pharmacogenetics is highly debated [15]. In rheumatology, only some genetic tests have been established, which are considered relevant to the dosing of some specific medications. For patients with unselected diagnoses, the PREPARE study showed that the concept of pharmacogenetic testing can be relevant to avoid side-effects [11]. As testing using the 12-gene pharmacogenetic panel also includes pharmacogenetic testing for the use of azathioprine (AZA), 6-mercaptopurine, thioguanine, rasburicase, tramadol and tacrolimus, the results of the PREPARE study may also apply to patients with rheumatic diseases.

The aim of this study was to assess the potential need and the performed genetic tests in a rheumatic out-patient cohort, especially for diagnostic, prognostic and pharmacogenetic purposes. The hypothesis was that genetic testing is relevant to more than 50% of rheumatic out-patients.

## 2. Patients and Methods

### 2.1. Study Design and Study Outcomes

Data from a prospective observational study with recruitment of consecutive out-patients (SolutionX) were retrospectively analyzed. The study design is cross-sectional at the time of the most recent visit. Physicians’ reports were included between 3 October 2017 and 9 January 2023. The setting is an out-patient clinic of rheumatology, serving patients needing secondary and tertiary levels of care.

The primary outcome was defined as the percentage of patients with a history of genetic testing, both with an inflammatory compared to a non-inflammatory rheumatic diagnosis. Current and potentially needed use of genetic testing according to specific diseases and patients’ characteristics (sex, age) were defined as secondary outcome parameters.

The STROBE criteria for observational cohort studies were applied [16]. The study was approved by the local Ethics Committee (AN2017-0041 370/4.18) and all patients gave informed and written consent before recruitment into the study.

### 2.2. Inclusion and Exclusion Criteria

All consecutive patients of the investigator were asked for their informed and written consent to participate in the SolutionX project. All patients were older than 17 years. Exclusion criteria were if the patients did not understand the German language or had any additional psychiatric disease disqualifying full understanding of consent.

### 2.3. Data Search

Microsoft Excel (Microsoft, version 16.70 (23021201), Redmond, WA, USA) was used for data management. An Excel file was prepared with all patients’ reports from the rheumatology outpatient clinic as identified in the local hospital information system (KIS). Data were pseudonymized before further analyses. Physicians’ reports were automatically searched for laboratory characteristics, genes, medications and diseases. The results were manually post-processed and checked for plausibility. The main diagnoses were subgrouped into the ‘inflammatory’ (1, 2, 3, 4, 5, 6 and 9) and the ‘non-inflammatory’ (0, 7, 8, 10 and 11) categories according to Appendix A Table A1. In the case of multiple diagnoses, inflammatory diagnoses were preferred to non-inflammatory diagnoses as main diagnosis. Genetic tests were grouped into diagnostic, prognostic, disease-related and comorbidity-related pharmacogenetic tests. Genetic testing for ‘diagnostic purposes’ applied to patients with a specific gene-related rheumatic disease (including SpA and psoriatic arthritis (PsA)), Behçet’s disease or rare diseases such as familial Mediterranean fever (FMF), cryopyrin-associated periodic syndrome (CAPS), familial cold autoinflammatory syndrome (FCAS), Muckle–Wells’ syndrome (MWS), neonatal onset multisystemic inflammatory disease (NOMID), chronic infantile neurologic cutaneous articular (CINCA) syndrome, familial cold autoinflammatory syndrome 2 (FCAS2), tumor necrosis factor receptor-associated periodic syndrome (TRAPS) or CARD14 mediated psoriasis (CAMPS)). ‘Prognostic purposes’ applied to patients with RA. ‘Therapeutic purposes’ were considered for patients with at least one drug with recommendation for genetic testing as listed in Appendix A Table A2. Some patients were receiving more than one of these drugs. ‘Disease-related’ medication applied for treatment of rheumatic diseases and pain (e.g., AZA, rasburicase, tacrolimus or tramadol), whereas ‘comorbidity-related’ medication included the drugs to treat patients’ comorbidities. To assess potential need for genetic testing, physicians’ reports were automatically screened for rheumatic diseases (Appendix A Table A1) and use of drugs (Appendix A Table A2).

### 2.4. Statistical Analyses

Both descriptive and further explorative statistics were applied. Statistical analysis was performed using Microsoft Excel. The median and interquartile range (IQR) for the median observation period, follow-up time and visits were used for description of the data as indicated. The mean value is provided for the parameter of age in patients’ characteristics. Comparisons between the categories of ‘inflammatory’ and ‘non-inflammatory’ diagnoses and between ‘female’ and ‘male’ were performed using the chi-square test. *p*-values < 0.05 are considered significant.

Patients without retrospectively confirmed diagnostic need for genetic testing may have been tested to exclude a diagnosis but could not be included into this analysis because of missing documentation of differential diagnoses. Age groups were also compared using the chi-square test. The age groups ‘10 to < 20’ and ‘90 to < 100 years’ were excluded because of the patients’ numbers being lower than 5 in one category. Bonferroni correction was used to adjust the probabilities (*p*-value) because of multiple testing.

## 3. Results

A total of 2490 patients were analyzed for the availability of genetic tests, with a median of 3 visits (IQR 1–7) per patient. The patients’ and diseases’ characteristics are shown in Table 1 according to the diagnostic categories (inflammatory or non-inflammatory) and groups (detailed in Appendix A Table A1). The main diagnoses were SpA, RA and other non-inflammatory diseases accounting for 24.6%, 14.4% and 15.3%, respectively, of all main diagnoses, and osteoarthritis/osteochondrosis, other inflammatory diseases, collagenoses/vasculitides (including patients with systemic lupus erythematosus, systemic sclerosis and myositis) and polymyalgia rheumatica (PMR)/giant cell arteritis (GCA) representing 13.8%, 8.1%, 6.1% and 6.1%, respectively.

### 3.1. Diagnosis-Related Genetic Testing

In this cohort, HLA testing was performed in 1281 patients (=51.4% of all patients). Data for patients with final diagnosis of SpA and *HLA-B5* for patients with final diagnosis of Behçet’s disease are shown in Table 2.

Subtyping of *HLA-B27* for the HLA-B27*06 and HLA-B27*09 variants was not performed in this routine setting [6]. HLA testing was performed in 665 patients (=26.7%) without diagnosis of SpA or Behçet’s disease in the observation period. Additionally, specific genetic tests were performed for genetic diseases with rare mutations (such as in fever syndromes).

### 3.2. Prognosis-Related Genetic Testing

HLA testing for DRB01*04 was performed in 0.4% of the RA patients. Such testing was performed in earlier times to assess the prognosis of these patients for patients’ education before introduction of potent treatments such as biological or targeted synthetic disease-modifying antirheumatic drugs. To the best of our knowledge, there are no other genetic tests currently recommended for prognostic purposes only in routine daily work.

### 3.3. Pharmacogenetic Testing

According to the PREPARE study, the patients’ medications were screened for a total of 28 drugs used by 697 (=28%) patients. The most frequently used drugs associated with potential benefit from pharmacogenetic testing are depicted in Table 3.

Out of these, 234 drugs in 224 patients are used for the treatment of a rheumatic disease. The most common disease-related medications were AZA (53.4%), tramadol (42.3%), tacrolimus (3.8%) and rasburicase (0.4%). From these 224 patients, pharmacogenetic testing was performed for specific mutations of thiopurine S-methyltransferase (TPMT) in 93 patients currently or formerly being treated with AZA (especially young women with diagnoses of SLE or specific vasculitides). There was no genetic testing for other medications related to the treatment of a rheumatic disease. No tests were performed for comorbidity-related therapeutic purposes.

Taken together, pharmacogenetic testing could be helpful for 28% of all patients. Out of these, testing would be helpful for drugs to treat rheumatic disease in 32.1% and for drugs to treat comorbidities in 76.9%.

### 3.4. Comparisons of Potentially Needed and Performed Genetic Testing

**Figure 1:**
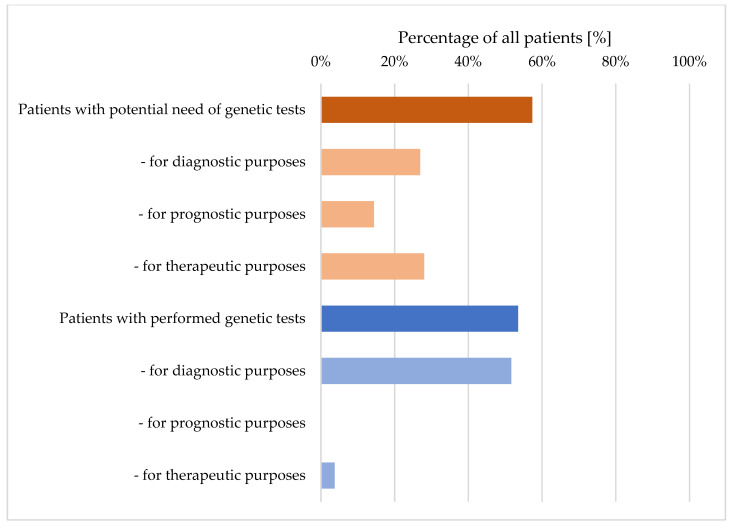
Comparison between percentages of patients with potential need for genetic tests (upper part) and of patients with actually performed genetic tests (lower part), including details of diagnostic, prognostic and therapeutic purposes.

Genetic testing was performed in 53.5% of all patients and was potentially needed in 57.3% of all patients (Figure 1). Thus, any genetic test was performed in 93.2% of the patients with potential need for genetic testing (Figure 1).

It is important to note that data regarding the potential need for genetic testing indicate a minimum of testing without considering the potential need for tests for diagnostic purposes to exclude differential diagnoses. This can be explained by the predefined method, i.e., that diagnostic genetic tests were assumed to be potentially needed only in patients with definite diagnosis of SpA, Behçet’s disease or any other genetic disease (such as fever syndromes). Therefore, concerning diagnostic purposes, there are more patients with performed tests than patients with potential need for genetic testing (51.6% vs. 26.3%, respectively; *p* < 0.001). In summary, 75.1% of patients with a positive genetic test performed for diagnostic purposes were finally diagnosed with the suspected disease. In detail, 81.2% of all HLA-B27 positive patients were diagnosed with SpA, 42% of all HLA-B51 positive patients were diagnosed with Behçet’s disease, 50% of MEVF positive patients were diagnosed with FMF and one patient with CARD-14 positivity was diagnosed with CAMPS.

When comparing patients of the inflammatory and the non-inflammatory categories, genetic tests were potentially needed for 1204 (=77.1%) inflammatory and for 224 (=24.1%) non-inflammatory patients (Table 4). Even more genetic tests were potentially needed, with 1544 and 232 genetic tests for the two categories, respectively, showing that some patients potentially need several genetic tests with different diagnostic, prognostic or pharmacogenetic purposes. Indeed, for the 926 and the 406 patients of the inflammatory and the non-inflammatory categories, respectively, slightly more genetic tests were actually performed (972 and 415 for the patients with inflammatory and non-inflammatory diagnoses, respectively). Taken together, there were more patients with a non-inflammatory diagnosis who were actually tested than patients for whom tests were potentially needed (181.3% versus 76.9%, respectively; *p* < 0.001), which can be considered a consequence of genetic testing to exclude differential diagnoses. Of note, patients were not genetically tested for comorbidity-related therapeutic purposes.

The number of patients with a potential need for genetic testing did not differ between female and male patients (57.2% vs. 57.6%, *p* = 0.87). Out of the potentially needed genetic tests, more tests were performed in the male group (98.1% vs. 90.9%; *p* = 0.004).

### 3.5. Age-Specific Analyses

**Figure 2:**
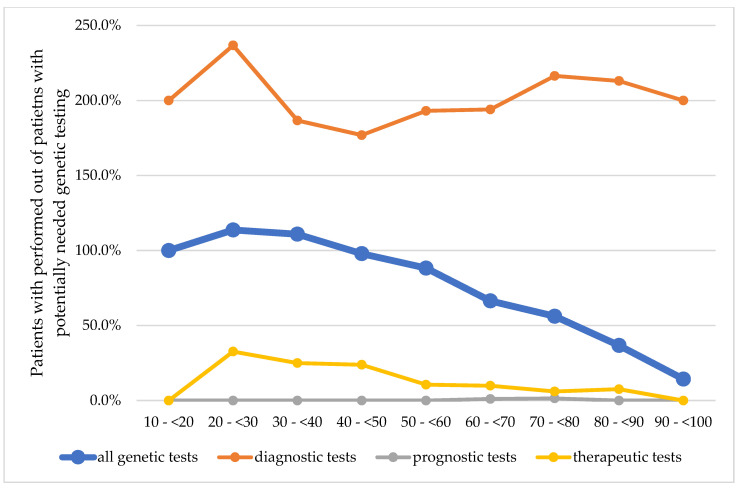
Comparison of patients with performed out of potentially needed genetic testing in all genetic tests and in the subgroups of diagnostic, prognostic and pharmacogenetic purposes on categories of age. A score of 100% indicates that all potentially needed tests were performed. Genetic testing for differential diagnoses was not included because of lack of documented reasoning.

The ratio of patients with performed tests to patients with a potential need for genetic testing decreased with increasing age, from 127.1% for 20 to <30-year-old patients to 46.1% for 80 to <90-year-old patients (Figure 2). The calculated potential need in general was higher with older age. The age groups 10 to <20 years and 90 to <100 years were excluded from the statistical analysis due to the small cohort sizes.

The potential for diagnostic genetic testing peaked in the age groups 30 to <50 years (34.2%) and decreased with increasing age. The potential for pharmacogenetic tests, however, was highest in the age group of 70 to <80 years (34.9%). While the potential for disease-related genetic tests decreased with increasing age (peak: 50.0% in age group 20 to <30), the potential for comorbidity-related gene tests was higher in older age groups (peak: 86.4% in age group 60 to <70 years). The number of patients with performed genetic tests also decreased with increasing age. Thus, the ratio of patients with performed versus potentially needed tests also confirmed this trend.

A consistently decreasing rate for performed genetic testing with increasing age can be shown for the inflammatory category of diseases (with 115.8% to 22.2% between ‘20 to <30 years’ and ‘90 to <100 years’, respectively). In the non-inflammatory category, there is an increasing rate of genetic testing with a peak of 257.4% in the age group ‘50 to <60 years’ and a subsequent decreasing rate (Table 5).

## 4. Discussion

With more than 55% of patients potentially benefiting from genetic testing in this rheumatic out-patient setting, the need for genetic testing is greater than expected, and testing was performed in 53.3% of all patients. Thus, the hypothesis that more than 50% of all rheumatic patients would need genetic testing was verified in this study. These data, however, still underestimate the need for genetic testing, as additional genetic testing for diagnostic purposes may be necessary and have been performed to support exclusion of possible differential diagnoses. Unfortunately, to the best of our knowledge, comparable data from other epidemiological studies are not available. For genetic testing concerning therapeutic purposes, data are scarce, and only data from the US shows clinically actionable pharmacogenetic drug prescriptions for 81% of adult rheumatic out-patients [17]. In the US cohort, the largest proportions of potentially impacted rheumatologic prescriptions were tramadol (47%), allopurinol (21%), AZA (17%) and celecoxib (8%) [17]. In contrast to the US data, data from our outpatient clinic show that the most-prescribed disease-related drugs with potential benefit from pharmacogenetic testing are AZA (53.4%) and tramadol (42.3%) (Table 3).

Pharmacogenetic testing was performed only in 93 out of 224 patients receiving a disease-related drug (=41.5%), urging the need for pharmacogenetic testing in routine clinical practice. These 93 tests were all TPMT tests recommended for dose adaptation of the immunosuppressive agent AZA (Table 3). With the use of AZA (and mercaptopurine and thioguanine), however, patients may also benefit from another test called nudix hydrolase 15 (NUDT15) (Table 3). According to the Clinical Pharmacogenetics Implementation Consortium (CPIC) guidelines, the degree of thiopurine intolerance is largely comparable between carriers of TPMT and NUDT15 decreased function alleles, although frequency of the mutated NUDT15 genes largely varies between ethnic groups, with 2% of patients having East Asian descent and 5% being Hispanic [18]. A full dose of mercaptopurine also poses a severe risk of prolonged hematopoietic toxicity in NUDT15-poor metabolizers and urges pre-emptive dose reductions of AZA. Therefore, it should be tested in Asians and Hispanics, whereas inherited TPMT deficiency is the primary genetic cause of thiopurine intolerance in Europeans and Africans [19]. So far, testing for mutations in NUDT15 is not routinely offered in this European clinical setting.

It is very important to avoid over-testing in clinical routine, and genetic tests are often only complementary, although they should be ordered only in the case of suspected disease, in order to not lead to incorrect diagnoses. However, in the non-inflammatory category, the assumed potentially needed diagnostic tests are fewer in number than the number of diagnostic tests performed (Table 5), as tests were assumed to be indicated only in patients with diagnosed diseases (e.g., for SpA, Behçet’s disease or any rare diseases). Thus, the non-inflammatory group appears to be ‘over-tested’, i.e., out of 232 possibly indicated tests, no diagnostic and prognostic indications can be confirmed because test indications for these categories are linked to differential diagnoses. Due to the retrospective assumption that only patients with the diagnosis have an indication for a diagnostic genetic test, there is a low potential need for these tests. This is one reason for the ‘over-testing’ in the non-inflammatory group. However, when focusing on the inflammatory group, these patients appear to be ‘under-tested’, and only 76.9% of potentially needed genetic tests were performed (Table 5). However, if obsolete prognostic tests for RA are excluded, the testing improves to 925 of 846 (=109.3%).

At present, widespread integration of pharmacogenetic testing into routine clinical practice appears to be complicated by several complex issues, including regulatory and reimbursement frameworks, evidence of clinical utility and clinical perspectives, practices and education. Thus, the rate of performed tests out of the potentially needed genetic tests consistently decreases between the ages of ‘20 to <30 years’ and ‘90 years to <100 years’ from 113.7% to as low as 14.3% (Figure 2). This finding is new and can be explained by the fact that genetic testing was not always available for routine testing in earlier years, and in the elderly, genetic testing for diagnostic purposes may not be needed anymore. The observed consistently decreasing rate for genetic testing with increasing age was also observed in the inflammatory group (‘20 to <30’ to ‘90 to <100 years’, from 115.8% to 22.2%). On the other hand, in the non-inflammatory category of diseases, the increased testing rate with a peak of 257.4% at age ‘50 to <60 years’ is likely caused by genetic testing to exclude SpA in differential diagnoses in patients with back pain, whereas the subsequent decreasing rate follows the lack of pharmacogenetic testing in the inflammatory category of diseases (Table 5). Overall, in this cohort, 75.1% of the patients with a positive genetic test developed the clinical disease, showing that HLA-B27, HLA-B51 and MEVF positivity alone may confirm an increased risk for the disease but not necessarily implies diagnoses of SpA, Behçet’s disease or FMF.

The clear strength of the study is its relatively large cohort of nearly 2500 consecutive rheumatic out-patients, especially chronic rheumatic patients with long-standing diseases from both subgroups of inflammatory and non-inflammatory pathophysiological background, which allowed further comparisons between these two categories with different therapeutic approaches. Also, the large study size allowed for comparisons of patients from different age groups, thus focusing on the higher prevalence of comorbidities in the aged population.

Most interestingly, a number of patients were without definite diagnosis. This could be improved via additional genetic testing, at least in single patients with inconclusive signs and symptoms. For example, a 24-year-old woman had genetic testing leading to diagnosis of a fever syndrome (CAPS) as a child. Now at age 24, however, additional testing for *HLA-B27* was performed to guide further diagnostic strategies for SpA. Such additional gene testing with important clinical consequences could have been avoided if the patient had already been screened and found to be positive at the first genetic testing. Also in this cohort, genetic analyses to diagnose vacuoles, E1 enzyme, X-linked, autoinflammatory and somatic (VEXAS) syndrome in patients with relapsing polychondritis could have been performed earlier if genetic data had been available at their first visit [20].

The main limitation of this study is its observational design with retrospective search of data from routine charts. The prospective design refers only to the recruitment of patients with their informed and written consent and not to structured data gathering. As data were acquired via manual search of the needed data, the time of ordering genetic tests was only available in a few patients and therefore not further assessed. Read-outs of structured data could be performed much faster, with more accuracy than manual searching. Structured data gathering for routine work would require detailed software support for several specific diagnoses in order to better document argumentation for the ordering of gene tests, depending on diagnostic, prognostic and therapeutic purposes. Thus, the rate of missed or falsely reported data could be further improved with the option of regular follow-ups over time. Also, the times of ordering may be interesting for planning future strategies on gene testing, including testing for pharmacogenetics in combination with disease-specific markers in rheumatic patients. Early testing may provide advantages compared to late testing, which may become more important with broader and cheaper availability of whole genome testing.

The data suggest that in the future, the use of genetic testing may be more complex than previously estimated. The role of pharmacogenetic testing is certainly underestimated, and the gene panel from the PREPARE study seems to be a good basis for further discussions [3,11]. Also, the study shows that some patients need more than one genetic test, either for diagnostic or pharmacogenetic purposes, supporting a strategy to combine DNA analyses with DNA screening purposes [3]. Together with clinical prescreening of the patients including assessment of typical signs and symptoms, DNA screening may also provide additional support to identify rare diseases. Negative gene results for diagnostic purposes may become relevant later for pharmacogenetic aspects. Thus, the potential of genetic testing is wide and will be used more and more in the future.

## 5. Conclusions

Genetic testing is routinely recommended and performed in more than 50% of all rheumatic out-patients. At present, diagnostic and pharmacogenetic indications are frequently performed, without need for risk assessments. With age, the rate of pharmacogenetic testing declines, although these tests could be helpful in patients with medications more and more needed to treat comorbidities in the elderly.

The practical implications of these data are multifold. In brief, genetic testing could benefit from combinations of diagnostic and pharmacogenetic analyses, thus avoiding multiple DNA preparations. In clinical practice, strategies for genetic testing for both diagnostic and pharmacogenetic purposes should be considered in cases of a suspected inflammatory rheumatic disease, especially in patients with comorbidities.

## Figures and Tables

**Table 1:** Patients’ characteristics in diagnostic subgroups (outlined in Appendix A Table A1).

Diagnostic Categories and Diagnoses	N [% of All]	Age [Years]	Sex [% Female]	Specific Lab Data [% of All Tests]
Inflammatory category	1561 (62.7)	58.2	63.1	
- RA	358 (14.4)	63.7	72.3	65.0% ACPA+
- pSpA/PsA	333 (13.4)	56.1	62.8	26.8% HLA-B27+
- axSpA	280 (11.2)	51.5	56.8	61.4 % HLA-B27+
- PMR/GCA	151 (6.1)	74.2	52.3	
- Collagenoses/vasculitides	154 (6.2)	52.7	77.9	70.2% ANA+, 22.5% ANCA+
- Cristal diseases	83 (3.3)	65.2	16.9	
- Other inflammatory diseases	202 (8.1)	50.2	71.3	
Non-inflammatory category	724 (29.1)	54.9	74.6	
- Osteoarthritis/osteochondrosis	344 (13.8)	62	77	
- Other diseases	380 (15.3)	48.5	72.4	
Neoplastic diseases	13 (0.5)	66.1	46.2	
Clinical and laboratory findings without diagnosis	192 (7.7)	51.4	68.8	

axSpA, axial SpA; ACPA, anti-citrullinated protein autoantibodies; ANA, antinuclear antibodies; ANCA, antineutrophil cytoplasmic antibodies; GCA, giant cell arteritis; PMR, polymyalgia rheumatica; RF, rheumatoid factor; PsA, psoriatic arthritis; pSpA, peripheral SpA.

**Table 2:** Genetic tests performed for diagnostic purposes in the total cohort and in those selected patients with the diagnoses listed in the second column of the table.

Tests	Diagnoses	All Patients [% Patients]	Selected Patients [% Patients]
Frequent genes			
HLA-B testing	SpA/Behçet’s	51.4	92.8
- HLA-B27	SpA	46.7	93.8
- HLA-B51	Behçet’s	2.9	80.0
Rare mutations			
*MEVF*	FMF	0.4	100
*NLRP3*	CAPS, FCAS, MWS, NOMID, CINCA syndrome	<0.1	100
*NLRP12*	FCAS2	<0.1	100
*TNFRS1A*	TRAPS	0.1	100
*CARD14*	CAMPS	<0.1	100
Genetic tests		52.6%	92.9%

CAMPS, CARD14 mediated psoriasis; CAPS, cryopyrin associated periodic syndrome; *CARD14*, caspase recruitment domain family member 14 gene; CINCA syndrome, chronic infantile neurologic cutaneous articular syndrome; FCAS, familial cold autoinflammatory syndrome; FMF, familial Mediterranean fever; HLA-B, major histocompatibility complex, class I, B; *MEVF*, gene for familial Mediterranean fever; MWS, Muckle–Wells’ syndrome; *NLRP3*, Nod-like receptor family pyrin domain containing 3; NOMID, neonatal onset multisystemic inflammatory disease; *NLRP12,* nucleotide-binding oligomerization domain, leucine-rich repeat-containing protein 12; SpA, spondyloarthritis; *TNFRS1A*, tumor necrosis factor receptor superfamily member 1A; TRAPS, tumor necrosis factor receptor-associated periodic syndrome.

**Table 3:** Frequency of drug use >2% in all patients, potential pharmacogenetic tests and number of performed tests (with percentages out of the patients treated with this drug). The marked medications (gray background) are medications related to rheumatic therapy; the other medications are used for the treatment of comorbidities.

Drug	Number of Patients [*n* (% of Total)]	Pharmacogenetic Testing	Performed Tests [*n* (%)]
Atorvastatin	168 (6.7)	*SLCO1B1*	0
AZA	125 (5.0)	*TPMT, NUDT15*	93 (74.4), 0
Tramadol	99 (4.0)	*CYP2D6*	0
Simvastatin	83 (3.3)	*SLCO1B1*	0
Estradiolvalerat	54 (2.2)	*F5*	0
Sertraline	52 (2.1)	*CYP2C19*	0

*SLCO1B1*, solute carrier organic anion transporter family member 1B1; *AZA*, azathioprine; *TPMT*, thiopurine S-methyltransferase; *NUDT15*, nudix hydrolase 15; *CYP2D6*, cytochrome P450 family 2 subfamily D member 6; *F5*, coagulation factor V; *CYP2C19*, cytochrome P450 family 2 subfamily C member 19.

**Table 4:** Comparison of potential needs and actually performed genetic testing shown for inflammatory non-infectious and non-inflammatory benign rheumatic disease categories. Differences between groups are tested using the chi-square test, with significance indicated if *p* < 0.05. After Bonferroni adjustments for multiple testing, results are shown with a local significance value of 0.006.

	Inflammatory [n (%)]	Non-Inflammatory [n (%)]	*p*
Patients with potential need for genetic tests	1204 (77.1)	224 (24.1)	<0.001
- for diagnostic purposes	656 (42.0)	0	
- for prognostic purposes	358 (22.9)	0	
- for therapeutic purposes	473 (30.3)	224 (24.1)	<0.001
Disease-related	174 (36.8)	50 (22.3)	<0.001
Comorbidity-related	354 (74.8)	182 (81.3)	-
Patients with performed genetic tests	926 (59.3)	406 (43.7)	<0.001
- for diagnostic purposes	883 (56.6)	403 (43.4)	<0.001
- for prognostic purposes	1 (0.1)	1 (0.1)	
- for therapeutic purposes	84 (5.4)	9 (1.0)	<0.001
Disease-related	84 (100)	9 (100)	
Comorbidity-related	0	0	

**Table 5:** Comparison of patients with potentially needed and performed genetic testing between the inflammatory and the non-inflammatory groups shown for age categories. Percentages of patients with potential need and performed genetic testing refer to the total cohort, whereas percentages of inflammatory and non-inflammatory groups refer to the number of patients with potential need or performed genetic testing, respectively. Differences between groups (excluding age groups ‘10 ≥ age < 20’ and ‘90 ≥ age < 100’) are tested using the chi-square test, with significance indicated by *p* < 0.05. After Bonferroni adjustment for multiple testing, results are shown with a local significance value of 0.003.

	Total Cohort [n (%)]	≥10 yrs <20 yrs [n (%)]	≥20 yrs <30 yrs [n (%)]	≥30 yrs <40 yrs [n (%)]	≥40 yrs <50 yrs [n (%)]	≥50 yrs <60 yrs [n (%)]	≥60 yrs <70 yrs [n (%)]	≥70 yrs <80 yrs [n (%)]	≥80 yrs <90 yrs [n (%)]	≥90 yrs [n (%)]	*p*
Patients’ numbers, total	2490 (100)	7	186	219	342	627	522	378	195	14	
Patients with potential need for genetic testing	1428 (57.3)	4 (57.1)	96 (51.6)	116 (53.0)	186 (54.4)	349 (55.7)	327 (62.6)	223 (59.0)	115 (59.0)	12 (85.7)	
- inflammatory group	1204 (77.1)	4 (100)	76 (79.2)	95 (81.9)	154 (82.8)	302 (86.5)	275 (84.1)	187 (83.9)	102 (88.7)	9 (75.0)	*
- non-inflammatory group	224 (24.1)	0 (0)	20 (20.8)	21 (18.1)	31 (16.7)	47 (13.5)	52 (15.9)	36 (16.1)	13 11.3)	3 (25.0)	*
Patients with performed genetic testing	1332 (53.5)	4 (57.1)	122 (65.6)	143 (65.3)	217 (63.5)	371 (59.2)	270 (51.7)	150 (39.7)	53 (27.2)	2 (14.3)	*
- inflammatory group	926 (59.3)	3 (75.0)	88 (72.1)	97 (67.8)	141 (65.0)	250 (67.4)	193 (71.5)	110 (73.3)	42 (79.2)	2 (100)	*
- non-inflammatory group	406 (43.7)	1 (25.0)	34 (27.9)	46 (32.2)	76 (35.0)	121 (32.6)	77 (28.5)	40 (26.7)	11 (20.8)	0 (0)	*
**Patients with performed out of potentially needed genetic testing**	93.2%	100%	127.1%	123.3%	116.7%	106.3%	82.6%	67.3%	46.1%	16.7%	*
- **inflammatory group**	76.9%	75%	115.8%	102.1%	91.6%	82.8%	70.2%	58.8%	41.2%	22.2%	*
- **non-inflammatory group**	181.3%	-	170.0%	219.0%	245.2%	257.4%	148.1%	111.1%	84.6%	-	*

*, *p* < 0.001

## Data Availability

The raw data supporting the conclusions of this article will be made available by the authors, if compatible with data security and safety.

## Appendix A

**Table A1:** Full list of diagnostic groups with synonyms used in physician’s reports.

	Main Diagnosis	Additional Diagnoses, Synonymes
1	Rheumatoid arthritis (RA)	palindromic rheumatism, juvenile idiopathic arthritis and systemic juvenile idiopathic arthritis (Still’s disease), late-onset RA (LORA)
2	Polymyalgia rheumatica ± large vessel arteritis (PMR/LVV), RS_3_PE	giant cell arteritis, Takayasu’s arteritis, aortitis, temporal arteritis RS3PE syndrome (=remitting seronegative symmetric synovitis with pitting edema)
3	Axial spondyloarthritis (axSpA)	ankylosing spondylitis
4	Peripheral spondyloarthritis (pSpA)	uveitis- and colitis-associated spondyloarthritis, juvenile idiopathic enthesitis associated arthritis psoriatic arthritis (PsA), reactive arthritis, synovitis-acne-pustulosis-hyperostosis-osteitis-syndrome (SAPHO-syndrome)
5	Collagenoses, other vasculitides (Coll./Vasc.)	systemic lupus erythematosus (SLE), antiphospholipid syndrome (APS), primary Sjögren’s syndrome eosinophilic granulomatosis with polyangiitis (EGPA), granulomatosis with polyangiitis (GPA), microscopic polyangiitis (MPA) Behçet‘s disease, leukocytoclastic and other vasculitides dermatomyositis, polymyositis systemic sclerosis, CREST-syndrome, mixed connective tissue disease, Sharp’s syndrome, undifferentiated collagenosis
6	Cristal diseases	gout, arthritis urica, chondrocalcinosis
7	Osteoarthritis, osteochondrosis	diffuse idiopathic skeletal hyperostosis
8	Non-inflammatory diseases	fibromyalgia, muscular dysbalances, myofascial pain syndrome chondropathia, disk prolaps/protrusion, neuroforaminal or spinal stenosis, scoliosis, Perthes disease, Scheuermann‘s disease Ehlers-Danlos syndrome Dupuytren’s disease, carpal tunnel syndrome osteopenia/osteoporosis, post traumatic injury, cerebral contusion vaccine-induced postthrombotic immune thrombocytopenia
9	Other inflammatory diseases	bursitis, enthesitis, osteitis symphysis pubica, tendinitis polychondritis, daktylitis, plantar fasciitis autoimmune hepatitis, colitis ulcerosa, Crohn’s disease, gluten sensitive enteropathy, autoimmune thyreopathy Hashimoto’s disease uveitis, retinitis, conjunctivitis, central retinal artery occlusion Eosinophilic alveolitis immune-related adverse events with polyarthritis, psoriasis without psoriatic arthritis, sarkoi-dosis, myasthenia membranous glomerulonephritis Fever syndromes (e.g., cryopyrin associated periodic syndrome)
10	Neoplastic diseases	benign tendosynovial giant-cell tumor leukemia/lymphoma, multiple myeloma, myelodysplastic syndrome, lymphomatoid papulosis mamma carcinoma, melanoma, pancreatic/pharyngeal/prostate carcinoma
11	Clinical and laboratory findings without diagnosis	elevated laboratory values without clinical relevance clinical signs and symptoms without clear diagnosis uncertainty of diagnosis

The category of ‘inflammatory’ diseases includes diagnoses from diagnostic groups 1, 2, 3, 4, 5, 6 and 9, and the category of ‘non-inflammatory’ diseases include diagnostic groups from 0, 7, 8, 10 and 11.

**Table A2:** List of trade names for drugs addressed in the PREPARE study which are available at this institution [11,21]. Marked drugs (grey background) are defined as drugs for the treatment of a rheumatic disease (‘disease-related’), whereas the other drugs are defined as drugs for the treatment of a non-rheumatic disease, i.e., a comorbidity.

Drug	Trade Names
5-Fluorouracil	5-Fluorouracil, Actikerall, Fluorouracil Accord, Tolak, Verrumal
6-Mercaptopurine	Mercaptopurin Silver, Puri, Xaluprine
Abacavir	Abacavir, Kivexa, Triumeq, Trizivir, Ziagen
Acenocoumarol	Acenocoumarol, Sintrom
Amitriptyline	Amitriptylin, Saroten
Aripiprazol	Aripiprazol, Abilify, Arileto
Atomoxetine	Atomoxetin, Atofab, Strattera
Atorvastatin	Atorvastatin, Atorvilbitin, Atozet, Caduet, Callator, Ezeato, Sortis, Trinomia
Azathioprine	Azathioprin, Azafalk, Immunoprin, Imurek, Jayempi
Capecitabine	Capecel, Capecitabin, Ecansya, Xeloda
Carbamazepine	Carbamazepin, Neurotop, Tegretol
Citalopram	Citalopram, Citalostad, Pram, Seropram
Clomipramine	Clomipramin, Anafranil, Clomicalm
Clopidogrel	Aclop, Clopidogrel, DuoPlavin, Grepid, Iscover, Plavix, Zyllt
Codeine	Codein, Resyl mit Codein
Doxepin	not available
Efavirenz	Efatriten, Efavirenz, Padviram, Stocrin, Sustiva
Escitalopram	Cipralex, Escitalopram, Pramulex
Estradiolvalerat	Progynova, Qlaira, Velbienne
Ethinylestradiol	Aliane, Alisma, Balanca, Belara, Bellgyn, Gynial, Danselle, Danseo, Daylina, Delia, Desofemine, Diane mite, Dienorette, Dienovel, Drosfemine, Drospifem, Elione Erlidona, EVRA, FemLoop, Flow, Gefemin, GinoRing, Gracial, Gynovin, Harmonette, Lenea, Levostrol, Liberel, Loette, Madinette, Madonella, Marvelon, Mayra, Meliane, Melleva, Mercilon, Microgynon, Midane, Minesse, Minulet, Mirelle, Motion, MyRing, NuvaRing, Peliette, Rigevidon, Seasonique, Selma, SetLona, Sibilla, Solvetta, Triodena, Valette, Varianta, Viola, Volina, Wave, Xyliette, Yasmin, Yasminelle, YAZ, Yirala, Yris
Flecianide	Amarhyton, Aristocor, Flecianid
Flucloxacillin	Floxapen, Flucloxacillin
Haloperidol	Haldol, Haloperidol
Imipramine	not available
Irinotecan	Irinotecan, Onivyde
Lamotrigin	Gerolamic, Lamictal, Lamotrigin
Metoprolol	Beloc, Lanoc, Metohexal, Metoprolol, Metoprolosuccinat, Seloken
Nortriptyline	Nortrilen, Nortriptylin
Oxcarbazepin	Trileptal, Oxcarbazepin
Paroxetine	Ennos, Paroxat, Paroxetin, Seroxat
Phenoprcoumon	Falithrom, Marcoumar, Phenoprocoumon
Phenytoin	Epanutin, Epilan-D
Pimozide	Orap, Pimozid
Propafenone	Propafenon, Rytmonorma
Rasburicase	Fasturtec, Rasburicase
Sertraline	Adjuvin, Sertralin, Tresleen
Simvastatin	Cholib, Ezesim, Simvastatin, Gerosim, Inegy, Nyzoc, SimEz, Simvastad, Simvatin, Zocord
Tacrolimus	Adport, Advagraf, Dailiport, Envarsus, Modigraf, Prograf, Protopic, Tacforius, Tacni, Tacrolimus
Tamoxifen	Ebefen, Nolvadex, Tamoxifen
Tegafur	Teysuno, Tegafur
Thioguanine	Thioguanin
Tramadol	Adamon, Lanalget, Noax, Skudexa, Tradocomp, Tradolan, Tralieve, Tramabene, Tramadog, Tramadol, Tramadolhydrochlorid, Tramadolor, Tramal, Tramastad, Tramundal, Tramvetol, Zaldiar
Venlafaxine	Efectin, Velostad, Venlafab, Venlafaxin
Voriconazole	VFEND, Voriconazol, Voriconazole
Warfarin	not available
Zuclopenthixol	Cisordinol, Zuclopenthixol

Not available: this drug is not available in Austria.

## References

[1] Eyre S., Orozco G., Worthington J. (2017). The genetics revolution in rheumatology: Large scale genomic arrays and genetic mapping. Nat. Rev. Rheumatol..

[2] Katsanis S.H., Katsanis N. (2013). Molecular genetic testing and the future of clinical genomics. Nat. Rev. Genet..

[3] Murray M.F., Giovanni M.A., Doyle D.L., Harrison S.M., Lyon E., Manickam K., Monaghan K.G., Rasmussen S.A., Scheuner M.T., Palomaki G.E. (2021). DNA-based screening and population health: A points to consider statement for programs and sponsoring organizations from the American College of Medical Genetics and Genomics (ACMG). Genet. Med..

[4] Kiltz U., Braun J., Becker A., Chenot J.F., Dreimann M., Hammel L., Heiligenhaus A., Hermann K.G., Klett R., Krause D. (2019). Langfassung zur S3-Leitlinie Axiale Spondyloarthritis Inklusive Morbus Bechterew und Frühformen. https://register.awmf.org/assets/guidelines/060-003l_S3_Axiale-Spondyloarthritis-Morbus-Bechterew-Fruehformen-2019-10.pdf.

[5] Khoury M.J., Feero W.G., Chambers D.A., Brody L.E., Aziz N., Green R.C., Janssens A.C.J., Murray M.F., Rodriguez L.L., Rutter J.L. (2018). A collaborative translational research framework for evaluating and implementing the appropriate use of human genome sequencing to improve health. PLoS Med..

[6] Khan M.A. (2013). Polymorphism of HLA-B27: 105 Subtypes Currently Known. Curr. Rheumatol. Rep..

[7] van der Helm-van Mil A.H.M., Huizinga T.W.J. (2008). Advances in the genetics of rheumatoid arthritis point to subclassification into distinct disease subsets. Arthritis Res. Ther..

[8] Wiik A.S. (2007). The Immune Response to Citrullinated Proteins in Patients With Rheumatoid Arthritis Genetic, Clinical, Technical, and Epidemiological Aspects. Clin. Rev. Allergy Immunol..

[9] Padyukov L. (2022). Genetics of rheumatoid arthritis. Semin. Immunopathol..

[10] Dedmon L.E. (2020). The genetics of rheumatoid arthritis. Rheumatology.

[11] Swen J.J., van der Wouden C.H., Manson L.E., Abdullah-Koolmees H., Blagec K., Blagus T., Böhringer S., Cambon-Thomsen A., Cecchin E., Cheung K.C. (2023). A 12-gene pharmacogenetic panel to prevent adverse drug reactions: An open-label, multicentre, controlled, cluster-randomised crossover implementation study. Lancet.

[12] Shekhani R., Steinacher L., Swen J.J., Ingelman-Sundberg M. (2020). Evaluation of Current Regulation and Guidelines of Pharmacogenomic Drug Labels: Opportunities for Improvements. Clin; Pharmacol. Ther..

[13] Malsagova K.A., Butkova T.V., Kopylov A.T., Izotov A.A., Potoldykova N.V., Enikeev D.V., Grigoryan V., Tarasov A., Stepanov A.A., Kaysheva A.L. (2020). Pharmacogenetic testing: A tool for personalized drug therapy optimization. Pharmaceutics.

[14] Caspar S.M., Schneider T., Stoll P., Meienberg J., Matyas G. (2021). Potential of whole-genome sequencing-based pharmacogenetic profiling. Pharmacogenomics.

[15] Luzum J.A., Petry N., Taylor A.K., Van Driest S.L., Dunnenberger H.M., Cavallari L.H. (2021). Moving Pharmacogenetics Into Practice: It’s All About the Evidence!. Clin. Pharmacol. Ther..

[16] Cuschieri S. (2019). The STROBE guidelines. Saudi J. Anaesth..

[17] Reid P., Danahey K., Lopez Velazquez M., Ratain M.J. (2022). Impact and applicability of pharmacogenomics in rheumatology: An integrated analysis Pharmacogenomics in rheumatology. Clin. Exp. Rheumatol..

[18] Relling M.V., Schwab M., Whirl-Carrillo M., Suarez-Kurtz G., Pui C.H., Stein C.M., Moyer A.M., Evans W.E., Klein T.E., Antillon-Klussmann F.G. (2019). Clinical Pharmacogenetics Implementation Consortium Guideline for Thiopurine Dosing Based on TPMT and NUDT15 Genotypes: 2018 Update. Clin. Pharmacol. Ther..

[19] Dr. Falk Pharma GmbH. Fachinformation Azafalk. https://aspregister.basg.gv.at/document/servlet?action=show&zulnr=1-31701&type=DOTC_FACH_INFO.

[20] Grayson P.C., Patel B.A., Young N.S. (2021). VEXAS syndrome. Blood.

[21] Bundesamt für Sicherheit im Gesundheitswesen and AGES Medizinmarktaufsicht “Arzneispezialitätenregister—Online Suche Arzneispezialitäten”. https://aspregister.basg.gv.at/aspregister/faces/aspregister.jspx.

